# Prevalence of Cardiovascular Disease Risk Factors in the Gambia: A Systematic Review

**DOI:** 10.5334/gh.827

**Published:** 2020-06-17

**Authors:** Robin Koller, Charles Agyemang

**Affiliations:** 1 Department of Public Health, Academic Medical Center, University of Amsterdam, Amsterdam Public Health Research Institute, Amsterdam, NL

**Keywords:** The Gambia, Cardiovascular disease, hypertension, smoking, obesity, diabetes

## Abstract

Cardiovascular disease (CVD) is increasingly becoming a major chronic disease burden in sub-Saharan Africa. The aim of this review was to provide an up-to-date overview on prevalence of CVD risk factors in the Gambia. The findings from seven included studies revealed that most CVD risk factors are very prevalent in the Gambia, with some specific groups in the population such as urban dwellers being more at risk. Obesity prevalence ranged from 2.3% to 11.7%, with rate being particularly high in urban women aged ≥35 years. Diabetes prevalence was 0.3%. Hypertension prevalence ranged from 18.3% to 29%. Prevalence of hypercholesterolemia ranged from 2.2% to 29.1%. Prevalence of smoking ranged from 16% to 42.2% in men. Prevalence of insufficient fruit and vegetable consumption, inadequate physical activity, and alcohol consumption was 77.8%, 14.6%, and 2.3%, respectively. These findings suggest urgent need for preventive measures and further research to prevent CVD in the Gambia.

## Introduction

Cardiovascular disease (CVD) is the main cause of morbidity and mortality worldwide [1,2] and the prevalence is increasing, with the greatest projected increases in low- and middle-income countries (LMICs) [3]. CVD is increasingly becoming a major health burden in sub-Saharan Africa (SSA) [1]. Between 1990 and 2013 the number of CVD deaths increased by 81% in this region [4]. In 2013, CVD caused 11.3% of deaths in SSA [4], of which the majority occur under the age of 70 years [5]. With infectious diseases still being a major health burden and the prevalence of CVD and other non-communicable diseases rising, SSA countries are now facing a double burden of disease [6].

The prevalence of CVD and its risk factors varies between SSA countries [7,8]. From 2003 to 2015, 33 member states in the WHO African Region conducted STEPS surveys to collect information on the status of major risk factors [9]. Prevalence rates derived from these surveys vary significantly. For example, in 2009 prevalence of smoking was significantly higher in Sierra-Leone (25.8%) than in Guinea (12.8%) [8]. In addition, prevalence of obesity was significantly higher in Gabon (15.9%) than in Malawi (4.4%) [8]. These variations may be related to differences in diets, cultural habits, and the levels of urbanisation and adoption of Western lifestyles [7,8].

Little is known about the prevalence of CVD and risk factors in the Gambia, one of the smallest African nations located on the West-African coast [10] with a population of 1.88 million in 2013 [11]. Almost half of the population is under the age of 15 years, and in 2016 life expectancy was estimated at 61 and 63 years for males and females, respectively [12]. The only available data on CVD in the Gambia, derived from a health and demographic surveillance system, show that stroke went from being the third most frequent cause of death among the 60+ age group in 1998–2001 to the top cause of death in 2005–2007 [13].

The majority of CVD is caused by risk factors that can be controlled, treated, or modified [14], such as hypertension, diabetes, elevated blood lipids, tobacco smoking, obesity, unhealthy diet, harmful use of alcohol, inadequate physical activity and psychosocial stress [7,8,9,14,15]. While systematic reviews on prevalence of CVD risk factors are available for several other SSA countries [16,17], CVD risk factor prevalence in the Gambia has not yet been systematically assessed. Mapping the prevalence of CVD risk factors within the Gambia can be an important base for policy on CVD prevention and treatment. Therefore, the main aim of this review was to fill the knowledge gap by providing an up-to-date overview on prevalence of CVD risk factors in the Gambia.

## Methods

### Literature search strategy

All papers on established CVD risk factors in adults (>15 years) in the Gambia, published between 1 January 1990 and 31 December 2018, and available in Pubmed database and African Journals Online database were systematically searched with the help of an experienced librarian. Additional searches of the Google Scholar database, as well as a manual search of references cited in the retrieved articles, were performed but did not lead to any additional inclusions. The search included terms on ‘Gambia’, ‘prevalence’, and each individual CVD risk factor – hypertension, diabetes, elevated blood lipids, overweight and obesity, tobacco smoking, high salt intake, alcohol consumption, insufficient fruit and vegetable consumption, and inadequate physical activity (Supplementary Table S1). No language restrictions were applied to the search.

### Screening and selection of articles

**Figure 1:**
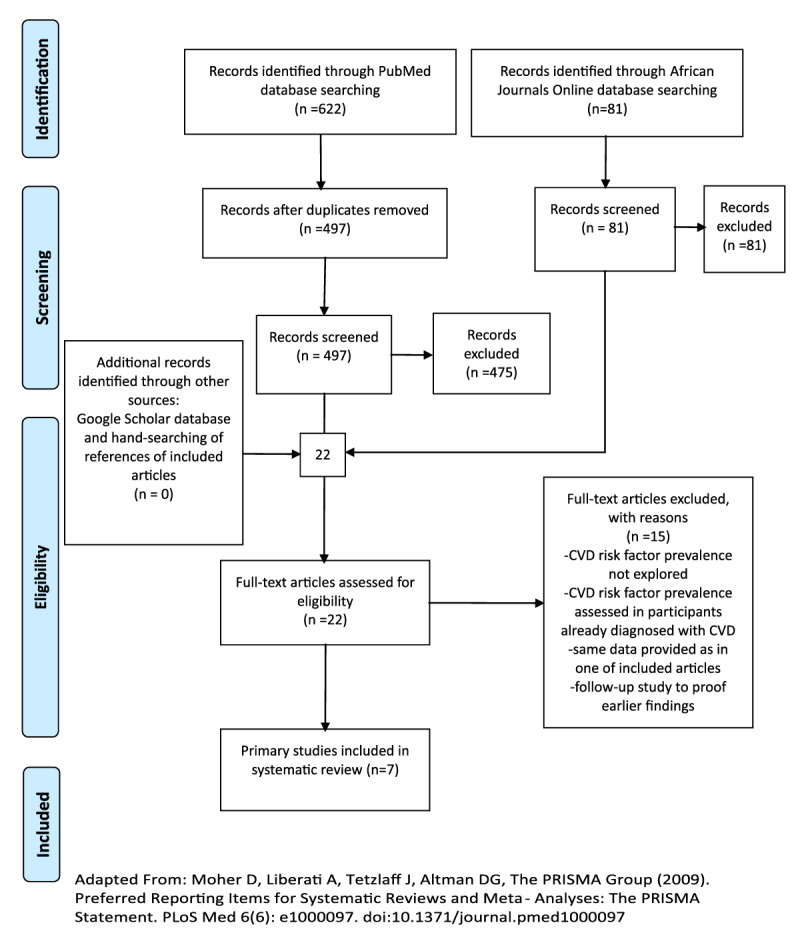
Study selection with flow diagram based on the PRISMA 2009 guidelines.

A total of 622 articles were identified through Pubmed. After removal of duplicates, there was a total of 497 articles. Title and abstract screening was performed by one reviewer (RK) using predetermined inclusion and exclusion criteria (Figure 1). After screening, 475 articles were excluded because they did not meet the predefined inclusion criteria. The African Journals Online database identified 81 articles, but after screening all 81 articles were excluded, because none assessed CVD risk factor prevalence. Consequently, the full-text of 22 articles was assessed for eligibility. Studies were included if they were population based, participants were aged ≥15 years, had a sample size ≥50 participants, were carried out in the Gambia and assessed prevalence of the aforementioned CVD risk factors. Fifteen articles were further excluded as they did not meet the above-mentioned inclusion criteria. Seven primary studies met the inclusion criteria and were included for data on prevalence of CVD risk factors [18,19,20,21,22,23,24]. The seven remaining studies were critically appraised using the risk of bias tool created by Hoy et al. for prevalence studies [25]. All 10 points of assessment were scored equally. A total score between zero and four was considered low risk of bias, 5–7 was moderate risk, and 8–10 was considered high risk of bias. Data from the included studies were extracted in a pre-formulated table (Table 1). The following data, if available, were extracted from the included studies: the first author’s surname, publication year, study site (urban and/or rural Gambia), sample size, response rate, sampling methods, and age-range. The applied definition of the CVD risk factors was extracted. Finally, prevalence data and, if available, the corresponding 95% confidence interval were extracted for the overall study population and for men and women separately if reported. One reviewer (RK) extracted data from individual studies, and the second reviewer (CA) re-evaluated the extracted data.

**Table 1:** Prevalence of cardiovascular risk factors in the Gambia.

Fist author surname and year	Site	a. Sample size b. response rate (%) c. sampling methods	Age range	Definition	Prevalence (%) (95% Cl)		

					Both sexes combined	Male	Female

Overweight and obesity							

Cham 2018 [18]	(Semi-)urban and rural	a. N = 3573 b. 77.9 c. Random sample*	25–64	Overweight: BMI ≥ 25 Obesity: BMI ≥ 30 Central obesity**	26.3 11.7 30.9	25.2 7.8 12.2	27.2 15.1 46.8
Van der Sande 1997 [19]	Urban and Rural	a. N = 6048 b. 94.9 c. Random sample	15–76+	Overweight: BMI 25–30 Obesity: BMI ≥ 30	8.1 2.3	NG NG	NG NG
Van der Sande 2000 [20]	Urban and Rural	a. N = 5389 b. Overall:78.1 Urban:67.7 Rural:87.1 c. Random sample	15–55+	Obesity: BMI ≥ 30	4.0^1^	Urban: 1.8 Rural: 0.1	Urban: 12.2 Urban ≥35 years: 32.6^1^ Rural: 1.1
Siervo 2006 [21]	Urban	a. N = 200 b. >90 c. Random sample	14–50	Overweight: BMI 25–30 Obesity: BMI ≥ 30 Central obesity	NG NG NG	Age 14–24: 0 Age 35–50: 6 Age 14–25: 0 Age 35–50: 6 Age 35–50: 8	Age 14–25: 10 Age 35–50: 34 Age 14–25: 6 Age 35–50: 50 Age 14–25: 12 Age 35–50: >80
**Hypertension**							

Cham 2018 [18]	(Semi-) urban and rural	a. N = 3573 b. 77.9 c. Random sample*	25–64	SBP ≥ 140 and/or DBP ≥ 90 mmHg and/or diagnosed hypertension	29 (26.6–31.8)	Overall: 27.7 (24.5–31.2) Age 25–34: 17.5 (13.9–21.8) Age 35–44: 26.3 (21.3–32.1) Age 45–54: 43.0 (35.8–50.4) Age 55–64: 53.4 (44.9–61.7) Urban: 23.9 (19.6–28.8) Semi-urban: 29.5 (22.1–38.2) Rural: 33.9 (29.6–38.6)	Overall: 30.5 (27.4–33.8) Age 25–34: 16.9 (44.9–61.7) Age 35–44: 33.6 (13.4–21.1) Age 45–54: 45.4 (29.3–38.1) Age 55–64: 60.4 (38.6–52.4) Urban: 26.1 (22.2–30.4) Semi-urban: 42.1 (29.7–55.5) Rural: 35.4 (31.7–39.3)
				Undiagnosed hypertension***	79 (74.5–82.2)	Overall: 86.0 (81.7–89.4) Age 25–34: 95.3 (88.4–98.2) Age 35–44: 93.7 (86.9–97.1) Age 45–54: 85.7 (76.5–91.7) Age 55–64: 61.3 (51.0–70.8) Urban: 88.0 (81.8–92.3) Semi-urban: 81.9 (64.1–92.0) Rural: 84.5 (77.5–89.6)	71.4 (65.2–76.9) Age 25–34: 88.2 (80.4–93.2) Age 35–44:73.0 (62.4–81.6) Age 45–54: 65.9 (55.7–74.9) Age 55–64: 54.7 (42.1–66.6) Urban: 69.1 (59.4–77.3) Semi-urban: 62.2 (54.2–69.6) Rural: 76.0 (66.0–83.8)
Van der Sande 2000 [20]	Urban and rural	a. N = 5389 b. Overall: 78.1Urban: 67.7Rural: 87.1 c. Random sample	15–55+	SBP ≥ 140 and/or DBP ≥ 90 mmHg	18.4	NG	NG
				SBP ≥ 140 and/or DBP ≥ 90 mmHg or on hypertension medication	Overall: 19.0 Urban: 20.3 Rural: 17.8	Urban: 22 Rural: 20.6	Urban: 16.9 Rural: 16.0
				SBP ≥ 160 and/or DBP ≥ 95 mmHg	7.1	NG	NG
				SBP ≥ 160 and/or DBP ≥ 95 mmHg or on hypertension medication	Overall: 7.6 Urban: 8.9 Rural: 6.9	Urban: 7.5 Rural: 7.6	Urban: 7.3 Rural: 6.3
Van der Sande 1997 [19]	Urban and Rural	a. N = 6048 b. 94.9 c. Random sample	15–76+	SBP ≥ 140 mmHg and/or DBP ≥ 90 mmHg	24.2	NG	NG
				SBP ≥ 160 and/or DBP ≥ 95 mmHg	9.5	NG	NG
Kobal 2004 [22]	Urban	a. N = 1997 b. NG c. Random sample	0–75	SBP ≥ 140 and/or DBP ≥ 90 mmHg	Overall: ^2^ Age 20+: 32.4^3^ Age 20–29: 13.2^3^ Age 30–39: 27.3^3^ Age 40–49: 44.8^3^ Age 50–59: 47.4^3^ Age 60–69: 83.8^3^ Age 70+: 77.3^3^	NG	NG
Jobe 2017 [23]	Rural	a. N = 2523 b. NG c. Random sample	≥18	SBP ≥ 140 and/or DBP ≥ 90 mmHg	Overall: 18.3(16.8–19.9) Age 18–29: 2.8(1.8–4.4) Age 30–39: 9.8(7.4–12.7) Age 40–49: 14.2(11.2–17.7) Age 50–59: 25.0(20.9–29.6) Age 60–69: 45.7(40.2–51.3) Age 70–79: 53.8(44.7–62.7) Age ≥ 80: 51.2 (36.5–65.6	Overall: 16.7 (14.1–19.6) Age 18–29: 1.0 (0.02–3.8) Age 30–39: 3.4 (1.1–10.1) Age 40–49: 9.6 (5.2–17.0) Age 50–59: 18.3 (12.6–25.8) Age 60–69: 35.2 (27.3–44.0) Age 70–79: 55.3 (41.1–68.8) Age ≥ 80: 57.9 (35.6–77.4)	Overall:18.9(17.2–20.9) Age 18–29: 3.6(2.2–5.7) Age 30–39: 11.1(8.4–14.5) Age 40–49: 15.4(12.1–19.7) Age 50–59: 28.3(23.0–34.1) Age 60–69: 52.7(45.5–59.7) Age 70–79: 52.9(41.2–64.2) Age ≥ 80: 45.8(27.5–65.4)
**Diabetes**							

Van der Sande 1997 [19]	Urban and Rural	a. N = 6048 b. 94.9 c. Random sample	15–76+	Fasting blood glucose ≥ 6.7 mmol/L after a positive glucosuria dipstick test	0.3	NG	NG
Van der Sande 2000 [20]	Urban and Rural	a. N = 2301 b. Overall: 78.1Urban:67.7Rural: 87.1 c. Random sample	≥35	2-hour 75-gram oral glucose tolerance test >10.0 mmol/L or currently on antidiabetic medication	NG	Urban: 7.9 Rural: 2.2	Urban: 8.7 Rural: 0.8
**Elevated blood lipids**							

Van der Sande 2000 [20]	Urban and Rural	a. N = 1075 b. Overall: 78.1Urban: 67.7Rural: 87.1 c. Random sample	≥35 years	Cholesterol > 5.2 mmol/L	NG	Urban: 12.5 Rural: 2.3	Urban: 29.1 Rural: 8.3
				Triglycerides > 1.8 mmol/L	NG	Urban: 4.0 Rural: 4.3	Urban: 4.0 Rural: 4.3
**Tobacco smoking**							

Cham 2018 [18]	(Semi-)urban and rural	a. N = 3573 b. 77.9 c. Random sample*	25–64	Current smokers	15.6	32.8	1.1
				Ex -smokers	5.2	10.8	0.6
				Never smoked	79.2	56.5	98.3
Van der Sande 2000 [20]	Urban and Rural	a. N = 5389 b. Overall: 78.1Urban: 67.7Rural: 87.1 c. Random sample	15–55+	Currently smoking	NG	Urban: 34.1 Rural: 42.2	Urban: 1.5 Rural: 5.9
				Ever smoked	NG	Urban: 46.9 Rural: 57.7	Urban: 3.0 Rural: 7.2
Siervo 2006 [21]	Urban	a. N = 200 b. >90 c. Random sample.	14–50	Currently smoking	NG	Age 14–25: 16 Age 35–50: 38	Age 14–25: 0 Age 35–50: 0
Jallow 2017 [24]	Urban and rural	a. N = 10289 b. 99 c. Random sample of secondary school students	12–20	Current smokers	Age 14–15: 3.6 Age 18–19: 5.5 Age 20: 5.7	NG	NG
				Ever smoked	Age 20: 19.8		
**Alcohol consumption**							

Cham 2018 [18]	(Semi-) urban and rural	a. N = 3573 b. 77.9 c. Random sample*	25–64	Ever consumed	2.3	3.7	1.1
**Insufficient fruit and vegetable consumption**							

Cham 2018 [18]	(Semi-) urban and rural	a. N = 3573 b. 77.9 c. Random sample*	25–64	<5 servings of fruit and vegetables per day	77.8	77.9	77.6
**Inadequate physical activity**							

	(Semi-) urban and rural	a. N = 3573 b. 77.9 c. Random sample*	25–64	<600 METS/week	14.6	12.0	16.7
Van der Sande 2000 [20]	Urban and Rural	a. N = 5389 b. Overall: 78.1Urban: 67.7Rural: 87.1 c. Random sample	15–55+	<0.5 day on their feet or leading a sedentary life	NG	Urban: 49.2 Rural: 48.1	Urban: 69.8 Rural: 58.0
Siervo 2006 [21]	Urban	a. N = 200 b. >90 c. Random sample	14–50	No physical activity or sport	NG	Age 14–25: 2 Age 35–50: 54	Age 14–25: 72 Age 35–50: 52

CI: confidence interval, BMI: body mass index, SBP: systolic blood pressure, DBP: diastolic blood pressure, MET: metabolic equivalent of task NG: Not given. ¹ Data available from another paper by van der Sande et al. published in 2001 [26] (same data source). ^2^ Includes data from participants under the age of 15 years, therefore not included. ^3^ Data available from another paper by Awad et al. published in 2014 [27] (same data source). *Based on secondary analysis of data from the WHO STEP survey 2010, conducted among 4111 randomly sampled participants. Restricted to non-pregnant participants with three valid blood pressure measurements. **Abdominal obesity: WC ≥ 90 cm in men and WC ≥ 80 cm in women. ***Proportion of hypertensives not aware of their condition prior to the survey.

## Results

### Characteristics of the study population

Some of the seven included studies provided data on one risk factor and some on multiple risk factors (Table 1) [18,19,20,21,22,23,24]. All studies were population-based cross-sectional studies. One study included only secondary school students [24]. Two studies distinguished between prevalence in urban and rural areas [18,20], four studies distinguished between males and females [18,20,21,23], and five studies provided data for different age groups [18,21,22,23,24].

The quality assessment performed on the included studies indicates low risk of bias in general. However, two studies did not report sampling methods such as response rate [22,23] and one study was based on convenient sampling [21].

### Prevalence of cardiovascular disease risk factors

#### Overweight and obesity

Four studies provided data on overweight and/or obesity prevalence [18,19,20,21]. Overweight was assessed in three studies [18,19,21], although different definitions were used. Two studies defined overweight as a BMI between 25 and 30 [19,21], and one study defined overweight as any BMI above 25 kg/m^2^ [18]. Overall prevalence of overweight ranged from 8.1% [19] to 26.3% [18]. Four studies assessed prevalence of obesity [18,19,20,21] and three of those studies reported on the overall prevalence [18,19,20], ranging from 2.3% [19] to 11.7% [18]. Three studies distinguished between males and females [18,20,21]. All of those studies showed a significantly higher prevalence of overweight and obesity in females than in males. One study distinguished between different age groups and found that prevalence of overweight and obesity increased with age and was especially high in women above the age of 35 [21]. One study distinguished between urban and rural obesity prevalence [20]. Obesity prevalence was found to be higher in urban areas (1.8% for males and 12.2% for females) than in rural areas (0.1% for males and 1.1% for females) [20]. Prevalence was especially high in urban women above the age of 35 (32.6%) [26].

#### Hypertension

Five studies provided data on hypertension prevalence [18,19,20,22,23]. All studies assessed prevalence of hypertension applying the current WHO criteria: systolic blood pressure (SBP) ≥ 140 and/or diastolic blood pressure (DBP) ≥ 90 mmHg [18,19,20,22,23]. Hypertension prevalence varied between studies, ranging from 18.3% [23] to 32.4% [22] of all participants. Two out of three studies that distinguished between males and females found a higher prevalence in females than in males [18,23], the third reported a higher prevalence in males [20]. Two studies distinguished between rural and urban prevalence [18,20] and reported conflicting results. The STEP survey found a significantly higher prevalence in rural areas [18], but van der Sande et al. found a higher prevalence in urban areas [20]. Three studies that distinguished between different age groups found that prevalence increases with age [18,22,23]. Cham et al. assessed the prevalence of undiagnosed hypertension and found that 79% of participants were unaware of their condition before the survey [18]. Prevalence of unawareness was higher in males than in females and in the younger age groups [18].

#### Diabetes

Two studies provided data on diabetes prevalence [19,20]. One study defined diabetes as fasting blood glucose ≥6.7 mmol/L (after a positive glucosuria dipstick test) [19], the other defined diabetes as blood glucose >10.0 mmol/L two hours after a 75g glucose load or currently on antidiabetic medication [20]. The first study reported an overall prevalence of 0.3% [19]. During the second study only adults above the age of 35 years were included, resulting in much higher numbers: 7.9% of urban males and 8.7% of urban females had diabetes, while in the rural areas this was 2.2% and 0.8%, respectively [20].

#### Elevated blood lipids

One study provided data on prevalence of elevated blood lipids in the Gambian population [20]. The study distinguished between urban and rural populations. Prevalence of elevated cholesterol (>5.2 mmol/L) was found to be higher in females than in males and higher in the urban population than in the rural population. In the urban population 12.5% of males and 29.1% of females ≥35 years had elevated cholesterol levels, while in the rural population this was 2.3% for males and 8.3% for females [20]. Prevalence of elevated triglycerides (>1.8 mmol/L) ranged from 4.0% in the urban area to 4.3% in the rural area for both males and females [20].

#### Tobacco smoking

Four studies provided data on prevalence of tobacco smoking [18,20,21,24]. Of these, three studies provided data on the prevalence of smoking in the overall population [18,20,21] and one assessed the smoking rate in secondary school students [24]. The prevalence of current smoking was 15.6% in the overall population [18]. Prevalence of current smoking ranged from 0% [21] to 5.9% [20] in females and from 16% [21] to 42.2% [20] in males. One study distinguished between rural and urban smoking prevalence and found a higher prevalence in the rural areas [20]. In the rural population prevalence rates of current smoking were 42.2% and 5.9% in males and females, respectively. In the urban population 34.1% of men were current smokers, compared to 1.5% of females [20]. Among secondary school students aged 20 years, 5.7% were current smokers and 19.8% ever smoked [24].

#### Alcohol consumption

One study provided data on the prevalence of alcohol consumption [18]. Overall prevalence was 2.3% [18]. Prevalence was higher in males than in females; 3.7% of males ever consumed alcohol, versus 1.1% of females [18].

#### Insufficient fruit and vegetable consumption

One study provided data on prevalence of insufficient fruit and vegetable consumption [18]. The percentage of participants who ate less than five servings of fruit and/or vegetables on average per day was 77.8% [18].

#### Inadequate physical activity

Data on prevalence of inadequate physical activity was provided in three studies [18,20,21]. All studies applied different definitions. The first study used ‘<600 MET-minutes per week’ [18], the second study used ‘<1/2 day on their feet or leading a sedentary life’ [20] and the third used ‘no engagement in physical activity or sport’ [21] to define inadequate physical activity. When applying the definition of less than 600 MET-minutes per week, the overall prevalence of inadequate physical activity was 14.6% [18]. The other two studies did not report overall prevalence [20,21]. All studies found a higher prevalence of inadequate physical activity in females than in males [18,20,21]. One study distinguished between different age groups and found that in the age group 35–50 years the difference between males and females was not statistically significant, but in the age group 14–25 years only 2% of males did not engage in physical activity or sports, compared with 72% of females [21]. Another study distinguished between urban and rural physical inactivity prevalence [20]. The prevalence of inadequate physical activity was higher in urban areas than in rural areas [20].

#### Salt intake

No studies provided data on daily salt intake in the Gambia.

## Discussion

### Key findings

Many of the reviewed CVD risk factors are prevalent in the Gambian population. Almost all of the studies, which distinguished between urban and rural areas, observed a higher prevalence of risk factors in urban than in rural areas. The prevalence of CVD risk factors vary by sex and age.

### Limitations

This review has some limitations. The availability of data on the prevalence of CVD risk factors in the Gambia was limited. Because a limited number of studies provided prevalence data on the various CVD risk factors, it was impossible to describe the trends over time. Additionally, there were variations in methods used in selecting participants, measurements, and definitions of the risk factors. The limited data, coupled with huge variations in the studies’ methods, made it impossible to carry out a meta-analysis. This could have led to differences in prevalence between studies. In addition, the reviewed studies were conducted in different years, varying from 1996 to 2016, which might have influenced the results of the studies. A study in 2001 has shown a great geographical variation in the prevalence of CVD risk factors within the Gambia, this could have led to variation in prevalence between studies [28]. Also, none of the studies provided age-standardised data, while there was a great variation in age structure between the different study populations. Furthermore, although the quality assessment performed on the included studies indicates low risk of bias, there were issues affecting some of the included studies including a lack of information on the sampling methods such as responsible rates [22,23] and use of convenient sampling method [21]. Moreover, the strength of the evidence for the various risk factors varied. For example, data on elevated blood lipids, alcohol consumption, and insufficient fruit and vegetable consumption were based on single studies, whereas risk factors such as overweight/obesity and hypertension were based on multiple studies. Lastly, some of the data were relatively old; for example, diabetes data were collected over two decades ago. These findings clearly suggest the need for up-to-date data to support current health policy initiatives and the intervention efforts in the Gambia. Despite these limitations, this current review provides very useful evidence that many CVD risk factors are prevalent in the Gambia.

### Discussion of key findings

Of all assessed CVD risk factors, insufficient fruit and vegetable consumption was most prevalent by far. This risk factor is estimated to cause about 11% of ischaemic heart disease deaths and about 9% of stroke deaths worldwide [29,30]. More than three out of four Gambian adults above the age of 25 ate less than the recommended five servings of fruit and/or vegetables on average per day. This may be explained by the fact that fruits and vegetables are seasonal in the Gambia and therefore become relatively expensive at some times of the year [31]. The prevalence of fruit and vegetable consumption in the Gambia is consistent with what is described in the rest of SSA, where prevalence of insufficient fruit and vegetable consumption ranges from 80% to over 90% in some countries [32].

The prevalence of smoking among Gambian men is among the highest in SSA [32,33] and is higher than the current prevalence among men in high-income countries (HICs) [34]. In West Africa, Sierra-Leone is the only country with a higher smoking rate [33]. Stricter control measures and increased awareness of smoking-related health problems have led to a reduction in tobacco consumption in HICs [35,36]. Tobacco companies are shifting their marketing operations to low- and middle-income countries (LMICs) [37], where health promotion programs to reduce smoking and legislation to discourage marketing are not or insufficiently regulated [33,37]. In the Gambia, cigarette advertisements and public smoking are banned; however, tobacco control policies require urgent strengthening [33]. Aggressive marketing, such as distributing free cigarettes in areas where youths gather, has been very effective in the Gambia. A study where determinants of tobacco smoking among Gambian youths were examined, reported that free cigarette offered by representatives of tobacco companies was mostly responsible for the difference between smoking and non-smoking youths [36].

The much lower prevalence of smoking in women compared to men could be explained by culture, gender role norms, and general expectations concerning gender-appropriate behaviour [33,38]. This same explanation could apply to the inequity in prevalence of alcohol consumption [39], which was the CVD risk factor with the lowest prevalence in the Gambia. In many SSA countries alcohol consumption is relatively frequent [32,39], but at least 90% of Gambians are Muslim [15] and are therefore often lifetime abstainers.

Other important differences in CVD risk factor prevalence between men and women were observed; obesity for example, mainly seems to be a problem for women above the age of 35, especially in urban areas, where 32.6% to 50% were found to be obese. Besides the physiological changes related to pregnancy and childbirth [32], this could also be due to cultural views on body size and attractiveness [32,40,41]. A study from 2006 on body image in the Gambia, describes that urban women between 35 and 50 years old were not worried about their body size until their BMI reached 27.8, whilst men of the same age and adults under the age of 35 years, started to be concerned at a BMI around 20 [41]. Urban women between the age of 35 and 50 also had a high rate (>80%) of central obesity [21], which is considered particularly detrimental [42]. In this same group of the population, a significantly higher prevalence of hypercholesterolemia and physical inactivity was found. The prevalence of elevated blood lipids is high in the adult SSA population [43]. A recent meta-analysis on the prevalence of dyslipidaemia in Africa found overall prevalence of total cholesterol >5.2 mmol/L and triglycerides >1.7mmol/L to be 23.6% and 16.5%, respectively [43]. Although the prevalence of dyslipidaemia in the Gambia was reported to be below this average, prevalence of elevated serum cholesterol in urban women above the age of 35 was 29.1% [20].

Gambian women are less likely to engage in sports and heavy manual labour, because of gender roles that exist [21,32]. Football, for example, is the most popular sport in the Gambia and is mainly played by men. This could explain why in the 14 to 25 years age group only 2% of males did not engage in physical activity or sports, compared with 72% of females [21]. When comparing the data from the WHO STEPS survey conducted in the Gambia [18] to the data from other SSA countries, it shows that many other SSA countries have a much higher prevalence of physical inactivity, measured in MET (metabolic equivalent of task) minutes per week than the Gambia [32]. However, the two other studies providing data on physical inactivity in the Gambia reported higher prevalence rates when applying other definitions for inadequate physical activity [20,21].

Hypertension, on the other hand is a risk factor without evident differences in prevalence between Gambian men and women. It is highly prevalent in all parts of society and prevalence clearly increases with age. The rate of hypertension in the Gambia is similar to what is observed in the rest of SSA [32,44]. Data from WHO STEPS surveys show that hypertension prevalence ranges from 20% to 40% in other SSA countries [32]. The burden of hypertension has been increasing continuously in SSA over the past few decades [45,46], SSA’s adult hypertension rate of around 30% lies above the global average and the average prevalence in HICs [44,47]. Although prevalence is high, a large proportion of those with hypertension remain undiagnosed, untreated, or inadequately controlled [44,47,48]. A meta-analysis from 2015 estimated the percentage of undiagnosed hypertension in SSA at 73%, with only 18% of hypertensives receiving treatment and around 7% having controlled blood pressure [44]. The Gambia is no exception; 79% of hypertensive STEPS survey participants were unaware of their condition prior to the survey [18], hence are at high risk for cardiovascular complications, such as stroke and heart disease.

Diabetes is another metabolic CVD risk factor that is largely undiagnosed in SSA (69.2% of cases in 2017, estimated by the International Diabetes Federation [49]). Prevalence of diabetes varies greatly between SSA countries; a review from 2011 reported prevalence of type 2 diabetes to be ranging from 1% in rural Uganda to 12% in urban Kenya [50]. Data from the Gambia is scarce, but diabetes was found to be prevalent in adults above the age of 35. In SSA diabetes prevalence seems to be peaking at a younger age, between the ages of 55 and 64, than in HICs where diabetes is most prevalent among the 75–79 years age group [49,51]. Because the published data on diabetes prevalence in the Gambia is from a study that was performed in 1997, current numbers are likely to be higher, as diabetes prevalence in SSA is increasing [49,50,52]. Because of the rapid growth and aging of the SSA population, changing lifestyle behaviours, combined with a genetic predisposition [52], the number of people living with diabetes in SSA is predicted to nearly double between 2010 and 2030 [53].

This review also provides evidence for inequities in prevalence of CVD risk factors between urban and rural areas in the Gambia. Urbanization is associated with dietary changes and sedentary lifestyle and therefore living in an urban environment increases the risk of developing CVD and risk factors [21,32,54]. SSA is urbanizing rapidly; in 2009 almost 40% of Africans lived in urban areas and this is projected to grow to 60% in 2050 [32]. This, together with ageing of the population will likely lead to growing prevalence of CVD and risk factors in SSA [32,55]. In the Gambia, most of the population (57%) is already concentrated around urban and peri-urban centres [15]. We found evidence that almost all CVD risk factors are more prevalent in urban Gambia, than in the rural population. However, for hypertension conflicting data were found [18,20] and smoking was an exception with a higher prevalence in rural areas [20]. This is an interesting finding, because urbanization, among other factors, is suggested to contribute to increased smoking rates in SSA and in most SSA countries prevalence of smoking is higher in urban areas than in rural areas [35,36]. This finding underlines that important differences exist between SSA countries and therefore the importance to assess the current CVD situation not only on a continental level, but on a national level as well.

## Conclusion

This review suggests that most CVD risk factors are very prevalent in the Gambia, with some specific groups in the population, such as urban dwellers, being more at risk. This high prevalence of CVD risk factors in the population is likely to lead to increasing prevalence of CVD. This suggests the urgent need for action to prevent CVD in the Gambia.

## Additional File

The additional file for this article can be found as follows:

10.5334/gh.827.s1 Supplementary Table S1. Keyword search terms.
