# Supplementary material for: Prevalence of Cardiovascular Disease Risk Factors in the Gambia: A Systematic Review
Source: Glob Heart. 2020 Jun 17;15(1):42. doi: 10.5334/gh.827 (PMC7427677; doi:10.5334/gh.827)
Supplement: Supplementary Table S1. — Keyword search terms. [file gh-15-1-827-s1.pdf]

Supplementary Table S1. Keyword search terms

Overweight and obesity

Search	Search terms	Hits
1	((Gambia[all fields]) OR (Gambia[mesh])) AND ((adipos*[all fields) OR (“body composition”[all fields]) OR (“body composition”[mesh]) OR (“Skinfold Thickness”[all fields]) OR (“Skinfold Thickness”[mesh]) OR (“body fat”[all fields]) OR (“high weight”[all fields]) OR (overweight[all fields]) OR (overweight[mesh]) OR (“body size”[all fields]) OR (“body size”[mesh]) OR (“body weight”[all fields]) OR (“body weight”[mesh]) OR (Obese[all fields]) OR (Obesity[all fields]) OR (Obesity[Mesh]) OR (“body mass”[all fields]) OR (BMI[all fields]) OR (QI[all fields]) OR (“Quetelet Index”[all fields]) OR (“Body Mass Index”[Mesh]) OR (“Waist Circumference”[Mesh]) OR (“Waist Circumference”[all fields]) OR (“abdominal fat”[all fields]) OR (“abdominal fat”[mesh]) OR (“waist-hip ratio”[all fields]) OR (“waist-hip ratio”[mesh]) OR (“waist hip ratio”[all fields]) OR (“waist-to-hip ratio”[all fields]))	278
2	#1 Limits: 1990/01/01 to 2018/12/31	214

Tobacco smoking

Search	Search terms	Hits
1	((Cigarette*[all fields]) OR (tobacco[all fields]) OR (“Tobacco Products”[Mesh]) OR (Smoking[all fields]) OR (Smoking[Mesh]) OR (smoke*[all fields])) AND ((Gambia[all fields]) OR (Gambia[mesh]))	38
2	#1 Limits: 1990/01/01 to 2018/12/31	32

Hypertension

Search	Search terms	Hits
1	((Gambia[mesh]) OR (Gambia[all fields])) AND ((hypertension[mesh]) OR (hypertension[all fields]) OR (tension*[all fields]) OR (“blood pressure*”[all fields]) OR (“blood pressure”[mesh]) OR (hypertensive[all fields]) OR (HTN[all fields]))	53
2	#1 Limits: 1990/01/01 to 2018/12/31	44

Diabetes

Search	Search terms	Hits
1	((Gambia[mesh]) OR (Gambia[all fields])) AND ((Insulin[all fields]) OR (Hyperinsulin*[all fields]) OR (Insulin[Mesh]) OR (glucose[all fields]) OR (glucose[mesh]) OR (Diabetic*[all fields]) OR (Diabetes[all fields]) OR ("Diabetes Mellitus"[Mesh]) OR ("Hemoglobin A, Glycosylated"[Mesh]) OR (HbA1c[all fields]) OR (“hemoglobin A1c”[all fields]) OR (“glycated hemoglobin”[all fields]) OR (“Glycosylated Hemoglobin”[all fields]) OR (glycohemoglobin[all fields]))	87
2	#1 Limits: 1990/01/01 to 2018/12/31	70

Insufficient fruit and vegetable consumption

Search	Search terms	Hits
1	((fruit*[all fields]) OR (vegetable*[all fields]) OR ("Potassium, Dietary"[Mesh]) OR (potassium[all fields])) AND ((gambia[mesh]) OR (gambia[all fields]))	26
2	#1 Limits: 1990/01/01 to 2018/12/31	19

Salt intake

Search	Search terms	Hits
1	(("Sodium Chloride"[Mesh]) OR (sodium[all fields]) OR (salt[all fields])) AND ((Gambia[all	35

	fields]) OR (Gambia[mesh]))	
2	#1 Limits: 1990/01/01 to 2018/12/31	25

Elevated blood lipids

Search	Search terms	Hits
1	((Gambia[mesh]) OR (Gambia[all fields])) AND ((cholesterol[mesh]) OR (cholesterol[all fields]) OR (blood lipid*[all fields]) OR (lipoprotein*[all fields]) OR (HDL[all fields]) OR (LDL[all fields]) OR ("Lipoproteins, LDL"[Mesh]) OR ("Lipoproteins, HDL"[Mesh]) OR (triglycerides [all fields]) OR (triglycerides[mesh]) OR (TGs[all fields]) OR (hyperlipidaemia[all fields]) OR (hyperlipidaemias[mesh]))	14
2	#1 Limits: 1990/01/01 to 2018/12/31	12

Inadequate physical activity

Search	Search terms	Hits
1	(("Motor Activity"[Mesh]) OR (“motor activity”[all fields]) OR (“physical activity”[all fields]) OR (exercise[all fields]) OR (labour[all fields])) AND ((gambia[mesh]) OR (gambia[all fields]))	237
2	#1 Limits: 1990/01/01 to 2018/12/31	192

Alcohol consumption

Search	Search terms	Hits
1	(("Alcohol Drinking"[Mesh]) OR ("Alcohol Abstinence"[Mesh]) OR (alcohol*[all fields])) AND ((Gambia[all fields]) OR (Gambia[mesh]))	16
2	#1 Limits: 1990/01/01 to 2018/12/31	14
